# Radiofrequency ablation using internally cooled wet electrodes in bipolar mode for the treatment of recurrent hepatocellular carcinoma after locoregional treatment: A randomized prospective comparative study

**DOI:** 10.1371/journal.pone.0239733

**Published:** 2020-09-28

**Authors:** Jae Won Choi, Jeong Min Lee, Dong Ho Lee, Jung-Hwan Yoon, Yoon Jun Kim, Jeong-Hoon Lee, Su Jong Yu, Eun Ju Cho

**Affiliations:** 1 Department of Radiology, Seoul National University Hospital, Seoul, Korea; 2 Department of Radiology, Seoul National University College of Medicine, Seoul, Korea; 3 Institute of Radiation Medicine, Seoul National University Medical Research Center, Seoul, Korea; 4 Department of Internal Medicine, Seoul National University Hospital, Seoul, Korea; 5 Department of Internal Medicine and Liver Research Institute, Seoul National University College of Medicine, Seoul, Korea; Yonsei University College of Medicine, REPUBLIC OF KOREA

## Abstract

**Objective:**

This study aimed to compare the efficacy between bipolar radiofrequency ablation (RFA), using twin internally cooled wet (TICW) electrodes, and switching monopolar RFA, using separable clustered (SC) electrodes, in the treatment of recurrent hepatocellular carcinoma (HCC) after locoregional treatment.

**Materials and methods:**

In this single-center, two-arm, parallel-group, randomized controlled study, we performed a 1:1 random allocation on eligible patients with recurrent HCC after locoregional treatment, to receive TICW-RFA or SC-RFA. The primary endpoint was the minimum diameter of the ablation zone per unit ablation time. Secondary endpoints included other technical parameters, complication rate, technical success and technique efficacy, and clinical outcomes.

**Results:**

Enrolled patients were randomly assigned to the TICW-RFA group (n = 40) or SC-RFA group (n = 37). The two groups did not show significant differences in the primary endpoint, the minimum diameter of the ablation zone per unit ablation time was 2.71 ± 0.98 mm/min and 2.61 ± 0.96 mm/min in the TICW-RFA and SC-RFA groups, respectively (p = 0.577). Total RF energy delivery (11.75 ± 9.04 kcal vs. 22.61 ± 12.98 kcal, p < 0.001) and energy delivery per unit time (0.81 ± 0.49 kcal/min vs. 1.45 ± 0.42 kcal/min, p < 0.001) of the TICW-RFA group were less than those of the SC-RFA group. No procedure-related death or major complications occurred. Technical success was achieved in all patients in both groups, and technique efficacy rates were 100% (46/46) in the TICW-RFA group and 95.0% (38/40) in the SC-RFA group (p = 0.213). The 1-year and 2-year cumulative LTP rates were 11.8% and 24.2%, respectively, in the TICW-RFA group, and 8.6% and 18.1%, respectively, in the SC-RFA group (p = 0.661).

**Conclusion:**

In this single-center randomized controlled study from a Korean tertiary referral hospital, TICW-RFA demonstrated similar therapeutic efficacy and safety profile for recurrent HCC after locoregional treatment compared with SC-RFA.

**Trial registration:**

ClinicalTrials.gov (NCT03806218)

## Introduction

Radiofrequency ablation (RFA) is currently recommended as an intended curative treatment for very early or early-stage hepatocellular carcinoma (HCC) in patients who are not surgical candidates according to guidelines from Europe, Asia, and North America [1–3]. Similarly, several previous studies also demonstrated that RFA could be used as a minimally invasive treatment modality for recurrent HCC [4–6]. However, a significant drawback of RFA has been the higher rate of local tumor progression (LTP) than that of surgical resection, for the treatment of HCC. This is probably due to the unreliable capability of RFA in producing sufficient ablative margin in a range of 5–10 mm [7–11]. Furthermore, in cases of recurrent HCC after locoregional treatment, LTP rates after RFA have been reported as being even higher than that in treatment-naïve HCC [12–15]. This could be attributed to tissue heterogeneity, indistinct tumor margin, or various off-target microenvironmental effects of previous treatment, leading to more invasive biological behavior of recurrent tumors [16–21]. Among them, heterogeneous tissue composition in recurrent tumors, including necrosis, fibrosis, adhesion, inflammation, and vaporized area after transcatheter arterial chemoembolization (TACE) or RFA may result in technical difficulty in delivering sufficient RF energy throughout the viable tumor, which ultimately may fail to kill tumor cells in the target tumor.

Until now, there have been various investigational approaches to efficiently create an ablation zone with a lethal thermal dose (> 60°C), enough to achieve sufficient ablative margin around the target tumor. These include modern high-powered RF devices with multiple electrodes, using switching monopolar [22, 23] or multipolar RFA [24, 25], a microwave ablation system [26–28], and combination with transarterial embolization or drugs [29–32]. Among them, in many institutes of Korea, including our hospital, two types of commercially available multiple-electrode systems have been used for the treatment of HCC: bipolar RFA, using twin internally cooled wet (TICW) electrodes (CWTN-T, RF Medical, Seoul, Korea) and a single-generator unit (M-3004, RF Medical); and switching monopolar RFA, using separable clustered (SC) electrodes with three active tips (Octopus, STARmed, Goyang, Korea) and a dual-generator unit (VIVA Multi, STARmed) [33]. Considering the tissue heterogeneity in recurrent tumors, we assumed that combined use of bipolar mode and saline perfusion in TICW-RFA, might provide additive value to the ablative efficiency by increasing current density between the electrode, and improving both electric and thermal conductivity [34].

Therefore, we conducted a randomized prospective comparative study between TICW-RFA and SC-RFA to compare their efficacy in the treatment of recurrent HCC after locoregional treatment.

## Materials and methods

### Study design

This single-center, two-arm, parallel-group, randomized controlled study was approved by the institutional review board of Seoul National University Hospital (#1502-105-652). After the approval by the institutional review board on April 13, 2015, participant recruitment and investigation were conducted at Seoul National University Hospital. All patients provided written informed consent at study enrollment. Patients underwent a 1:1 random assignment to the TICW-RFA or SC-RFA group. We applied a blocked randomization method with mixed block sizes 4 and 6, using a web-based allocation table, generated ahead of the study and managed by our institution’s medical research collaboration center. Randomization was stratified by the length of the active tip of the RFA electrode (2 cm or 2.5 cm), as the length of the active tip is determined according to the size of the index tumor. Study participants and those assessing outcomes were blinded to group assignment.

Although public registration before participant recruitment is recommended for a clinical trial, this study was initially recorded at our institutional clinical study database. We additionally registered our study at ClinicalTrials.gov (NCT03806218) for publication after it ended. The authors confirm that all ongoing and related trials for this intervention are registered. RF Medical Co., Ltd. (Seoul, Korea) provided a research grant for this study. The authors had complete control of the data and information at all times.

### Patients

**Fig 1:**
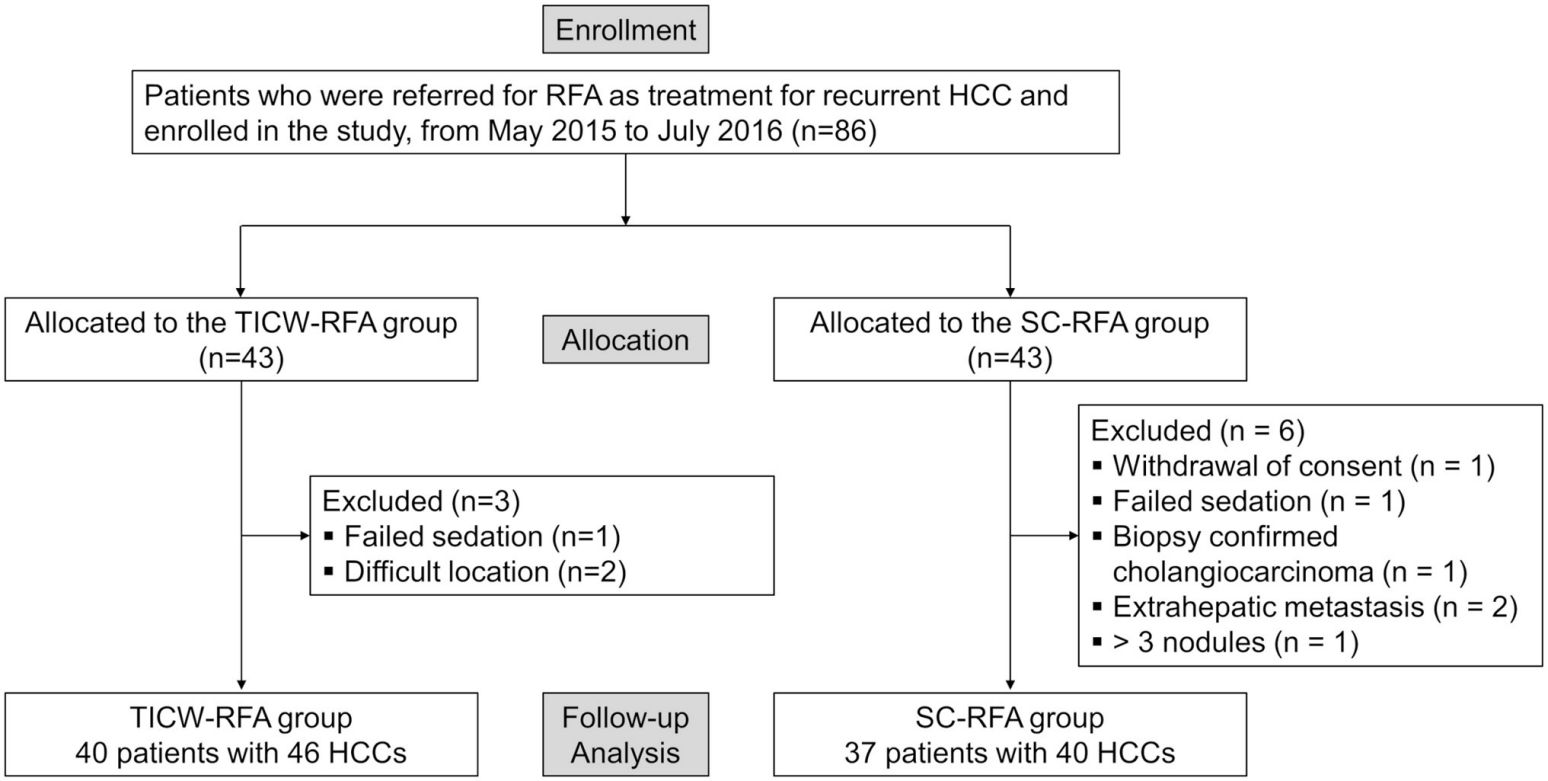
Flow chart of the study population.

From May 19, 2015, to July 4, 2016, one of the authors (J.M.L.) recruited those who met the following inclusion and exclusion criteria among patients who were referred to our department for RFA, as treatment for intrahepatic recurrent HCC after locoregional treatment (Fig 1). Inclusion criteria were as follows: (a) age 20 to 80 years, (b) radiologic or pathologic diagnosis of intrahepatic recurred HCC, including both LTP and intrahepatic distant recurrence (IDR) after locoregional treatment, and (c) HCC nodules measuring 1 cm or larger, but smaller than 5 cm. Exclusion criteria were as follows: (a) more than three HCC nodules, (b) tumors with major vascular invasion or abutment to the central portal or hepatic vein with a diameter >5 mm, (c) extrahepatic metastasis, (d) Child-Pugh class C, and (e) severe coagulopathy (platelet cell count of less than 50,000 cells/mm^3^ or prothrombin time international normalized ratio (PT-INR) prolongation of more than 50%).

### RFA procedure

One experienced radiologist (J.M.L.), with 20 years of experience in RFA, conducted all RFA procedures on an inpatient basis, assisted by one radiology fellow or resident. Evaluation of feasibility and planning of RFA procedures were performed based on pre-procedural CT or MRI studies and fusion imaging techniques between real-time US imaging and reference CT or MRI images [35]. Before percutaneous electrode insertion, intravenous conscious sedation and local anesthesia were induced. Throughout the procedure, patients underwent continuous monitoring of vital signs, electrocardiography, and oxygen saturation levels.

**Fig 2:**
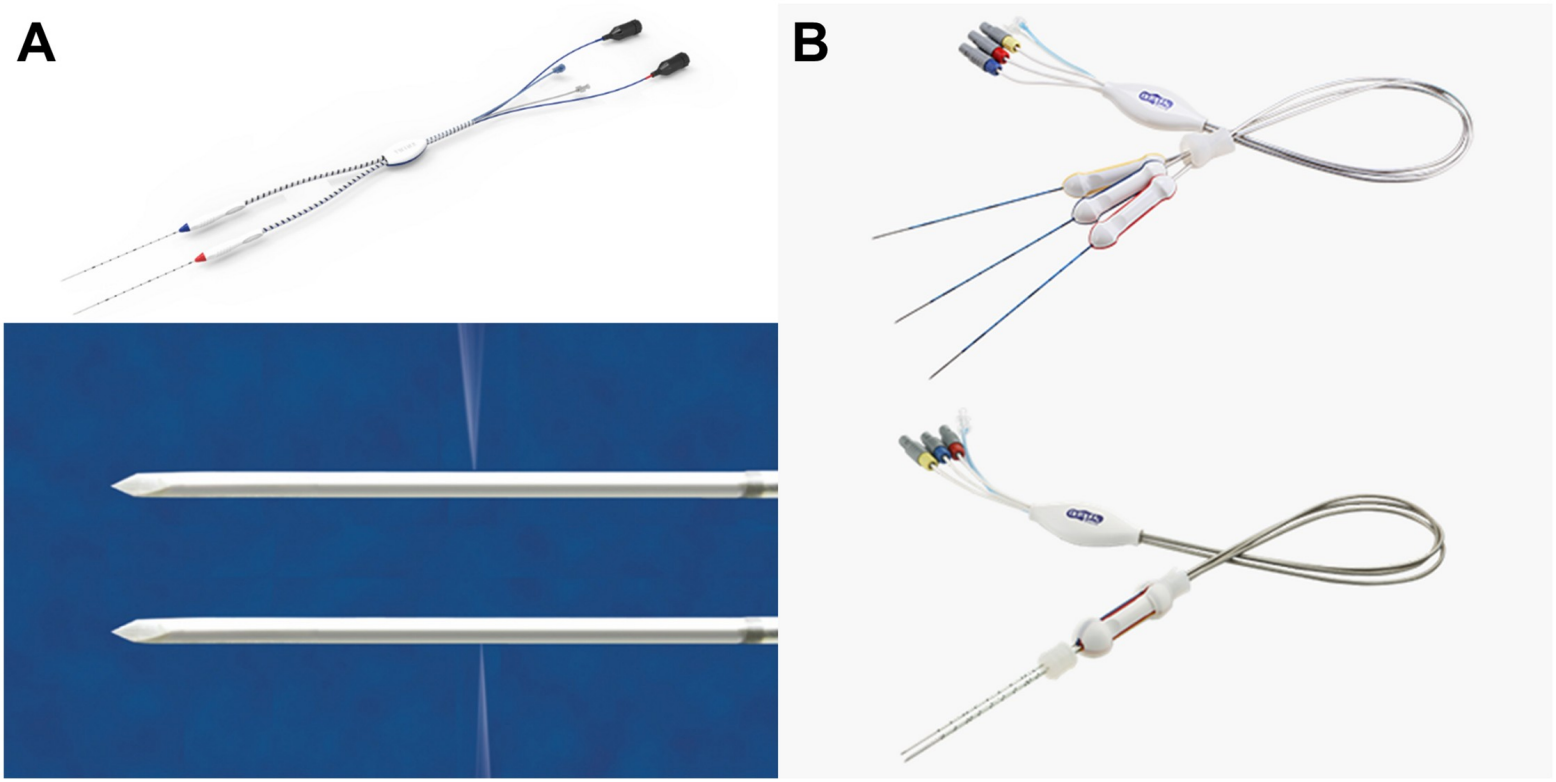
Photographs of (A) twin internally cooled wet (TICW) electrodes (CWTN-T, RF Medical, Seoul, Korea) and (B) separable clustered (SC) electrodes with three active tips (Octopus, STARmed, Goyang, Korea).

In both the TICW-RFA and SC-RFA groups, RFA was performed based on the ablation protocols used in routine clinical practice. In the TICW-RFA group, bipolar RFA was performed using TICW electrodes (Fig 2A; CWTN-T, RF Medical, Seoul, Korea) and a single-generator unit (M-3004, RF Medical). Chilled 0.9% isotonic saline was circulated inside the electrode, 99% for cooling the electrode, and 1% for saline infusion into the surrounding tissue [36]. The SC-RFA group underwent switching monopolar RFA, using SC electrodes with three active tips (Fig 2B; Octopus, STARmed, Goyang, Korea) and a dual-generator unit (VIVA Multi, STARmed). The details of the equipment and working process of the RFA systems were the same as described in previous studies [36–39].

The operator chose the length of the active tip, based on the size of the index tumor. In general, the operator used electrodes with a 2-cm active tip for a tumor smaller than 2 cm, and those with a 2.5-cm active tip for a larger one [39]. The fusion imaging technique was applied for initial targeting of the index tumor, to improve tumor visibility and overall technical feasibility [40], and also for intraprocedural monitoring of the ablation [35, 41]. If needed, the operator instilled a 5% dextrose solution into the perihepatic space for artificial ascites, to improve the sonic window, or to prevent adjacent organ injury while treating subcapsular tumors [42].

### Evaluation of procedure and follow-up

Multiphasic contrast-enhanced CT studies were conducted immediately after all the RFA procedures for assessment of ablation size, post-procedural complications, and technical success based on the reporting criteria suggested by the International Working Group on Image-guided Tumor Ablation [43]. The zone of ablation was defined as the non-enhancing hypoattenuating area on the portal phase CT [44], and diameters and volume of the ablation zone were measured. Assuming that the ablation zone was spherical as described in the previous study [22], the ablation volume and the effective ablation volume were measured as follows:

$$Ablation\;volume\;=\;\frac{\pi \times Dmax\times Dmin\times Dv}{6}$$

$$Effective\;ablation\;volume\;=\;\frac{\pi \times {Dmin}^{3}}{6},$$

where Dmax and Dmin are the longest and shortest diameters of the ablation zone on the axial image with the largest ablation area, and Dv is the longest vertical diameter of the ablation zone on the coronal reconstructed image. In addition to size measurements, ablation time and amount of energy delivery were also recorded.

We evaluated major complications, such as post-procedural events that extended the amount of care or prolonged the stay in the hospital, according to the guidelines of the Society of Interventional Radiology [43, 45].

Based on the standardization of terminology and reporting criteria proposed by the International Working Group on Image-guided Tumor Ablation [43], we defined technical success as an ablation that completely covered the index tumor with an ablative margin greater than or equal to 5 mm at the immediate post-procedural CT. Any irregular or nodular peripheral enhancement at the ablation margin, was regarded as indicating an unablated residual tumor and a treatment failure [46]. Patients with an initially unsuccessful RFA underwent an additional ablation in less than 24 hours during the same hospital stay. Technique efficacy was evaluated as complete coverage of the ablation of the index tumor, with no nodular arterial enhancement at the ablation zone on a 1-month follow-up CT or MRI scan [37, 43, 47].

For 2 years after RFA, patients underwent contrast-enhanced CT or MRI every 3 months for the detection of LTP as well as IDR and extrahepatic metastasis (EM) [37, 43]. LTP was defined as the appearance of tumor foci at the periphery of the ablation zone, after at least one contrast-enhanced follow-up study had recorded technical success and technique efficacy according to imaging criteria [43]. Recurrence-free survival (RFS) time was defined as the duration of the follow-up until LTP, IDR, EM, or death occurred. Moreover, among recurrence cases, we defined aggressive intrasegmental recurrence (AIR) as the simultaneous multinodular (three or more) recurrence or infiltrative tumor recurrence in the treated segment of the liver, at least 6 months after a disease-free state following RFA [48].

### Outcomes

The primary endpoint was Dmin per unit time. Secondary endpoints were as follows: other technical parameters including size of the ablation zone, ablation time, and energy delivery; complication rate; technical success; technique efficacy; and clinical outcomes including LTP rates, LTP-free survival, and RFS.

### Statistical analysis

We calculated the sample size using an approximation of the difference in the primary endpoint between the two groups, a two-sided type I error of 0.05, and a power of 0.8. We estimated that the difference in Dmin per unit time between the two groups would be 0.63 based on a previous study comparing the bipolar RFA and the switching monopolar RFA [37]. The minimum sample size was calculated to be 41 patients in each group, and assuming the drop rate of 5%, we decided to enroll 43 patients for each group. Technical parameters, technical success, technique efficacy, and LTP rates were analyzed with per-nodule data. Complications and other clinical outcomes were analyzed with per-patient data. Categorical variables were compared using the chi-squared test or Fisher’s exact test, as appropriate. Continuous variables that did not pass the Shapiro-Wilk normality test were compared using the Mann-Whitney test. Other continuous variables were compared using the independent t-test or Welch test, as appropriate. We used the Kaplan-Meier method for survival analysis and the log-rank test for assessing differences between the survival curves. We performed multivariate Cox proportional hazards regression analysis using the group allocation and the baseline characteristics to evaluate the relative risk factors associated with LTP and RFS. A p-value of less than 0.05 was considered a significant difference. Statistical analyses were conducted using MedCalc Statistical Software version 17.6 (MedCalc Software bvba, Ostend, Belgium).

## Results

### Patients

From May 19, 2015, to July 4, 2016, 86 patients were initially included in the study and underwent 1:1 randomization, with 43 patients in the TICW-RFA and 43 patients in the SC-RFA group. Among 86 patients, 9 patients were excluded from the study: withdrawal of consent (n = 1), difficult location (abutting diaphragm) (n = 2), failed sedation (n = 2), biopsy-confirmed cholangiocarcinoma (n = 1), diagnosed extrahepatic metastasis on immediate post-RFA CT (n = 2), and more than 3 nodules detected on the day of procedure (n = 1). The final study population was 77 patients: 40 patients with 46 nodules treated with TICW-RFA and 37 patients with 40 nodules treated with SC-RFA (Fig 1). The baseline characteristics of the study population are shown in Table 1. The proportion of LTP in the recurred target tumors did not show a significant difference between the TICW-RFA group and the SC-RFA group (78.3% vs. 70.0%, respectively, p = 0.384). We defined early and late recurrences as those that had recurred within 12 months or after 12 months after the previous locoregional treatment [49]. The proportion of early recurrences was not significantly different between the TICW-RFA group and the SC-RFA group (72.5% vs. 56.8%, respectively, p = 0.148). Both groups underwent various previous locoregional treatments, including RFA, TACE, and percutaneous ethanol injection therapy (PEIT), and the combination of different modalities showed the highest proportion in both groups. The TICW-RFA group had a higher PT INR (1.12 ± 0.09 vs. 1.06 ± 0.08, p = 0.002) and a trend toward less subcapsular tumors (23.9% vs. 42.5%, p = 0.068) than the SC-RFA group. Otherwise, there were no significant differences between the two groups concerning demographic features, tumor size and number, serum AFP level, and liver function.

**Table 1:** Baseline characteristics of the study population.

	TICW-RFA (n = 40^†^)	SC-RFA (n = 37^†^)	P-value
Age	63.8 ± 9.9	64.2 ± 10.1	0.883
M/F ratio	34/6	31/6	0.884
Single HCC, %	87.5 (35/40)	91.9 (34/37)	0.713
Size^‡^, cm	1.57 ± 0.54	1.72 ± 0.78	0.496
Used active tip, %			0.906
2 cm	50.0 (20/40)	48.6 (18/37)	
2.5 cm	50.0 (20/40)	51.4 (19/37)	
Subcapsular location^‡^, %	23.9 (11/46)	42.5 (17/40)	0.068
AFP, ng/ml	20.4 ± 34.8	77.1 ± 218.8	0.625
Child-Pugh class, %			0.202
A	87.5 (35/40)	97.3 (36/37)	
B	12.5 (5/40)	2.70 (1/37)	
Albumin	3.91 ± 0.52	4.05 ± 0.41	0.184
Bilirubin	0.89 ± 0.59	0.69 ± 0.48	0.069
PT INR	1.12 ± 0.09	1.06 ± 0.08	0.002
Platelet, × 1000/mm3	131.7 ± 71.4	135.3 ± 43.0	0.169
Type of recurrence^‡^, %			0.384
LTP	78.3 (36/46)	70.0 (28/40)	
IDR	21.7 (10/46)	30.0 (12/40)	
Onset of recurrence, %			0.148
Early (< 12 mo)	72.5 (29/40)	56.8 (21/37)	
Late (≥ 12 mo)	27.5 (11/40)	43.2 (16/37)	
Previous treatment modality, %			
RFA	5.0 (2/40)	13.5 (5/37)	
TACE	45.0 (18/40)	32.4 (12/37)	
PEIT	0.0 (0/40)	2.7 (1/37)	
≥ 2 modalities	50.0 (20/40)	51.4 (19/37)	

Note.—LTP = local tumor progression, IDR = intrahepatic distant recurrence.

^†^Number of patients,

^‡^Type of recurrence, tumor size, and frequency of subcapsular tumors were measured on a per-nodule basis. Plus-minus values are means ± standard deviations.

### Technical parameters

The TICW-RFA group and the SC-RFA group did not show significant differences in the primary endpoint, Dmin per unit time (2.71 ± 0.98 mm/min vs. 2.61 ± 0.96 mm/min, p = 0.577), or in any other ablation size-related variables (Table 2) or ablation time (13.92 ± 4.96 min vs. 15.60 ± 6.80 min, p = 0.441). In the TICW-RFA group, total RF energy delivery (11.75 ± 9.04 kcal vs. 22.61 ± 12.98 kcal, p < 0.001) and energy delivery per unit time (0.81 ± 0.49 kcal/min vs. 1.45 ± 0.42 kcal/min, p < 0.001), were smaller than in the SC-RFA group.

**Table 2:** Comparison of technical parameters between TICW-RFA and SC-RFA groups.

	TICW-RFA (n = 46^†^)	SC-RFA (n = 40^†^)	P-value
Dmin/time, mm/min	2.71 ± 0.98	2.61 ± 0.96	0.577
Dmin/energy, mm/kcal	4.37 ± 2.65	1.95 ± 0.91	< 0.001
Dmin, cm	3.40 ± 0.66	3.58 ± 0.83	0.254
Dmax, cm	4.84 ± 0.92	5.29 ± 1.22	0.057
Dv, cm	3.80 ± 1.03	4.21 ± 1.20	0.169
Ablation time, min	13.92 ± 4.96	15.60 ± 6.80	0.441
Energy, kcal	11.7 ± 9.0	22.6 ± 13.0	< 0.001
Energy/time, kcal/min	0.81 ± 0.49	1.45 ± 0.42	< 0.001
Ablation volume, cm^3^	35.24 ± 18.50	46.12 ± 31.15	0.222
Ablation volume/time, cm^3^/min	2.64 ± 1.22	3.08 ± 1.75	0.387
Effective ablation volume, cm^3^	22.77 ± 12.26	28.02 ± 21.31	0.637
Effective ablation volume/time, cm^3^/min	1.71 ± 0.87	1.88 ± 1.32	0.962

Note.—Dmax, Dmin = longest and shortest diameters of the largest ablation zone on axial plane, Dv = longest vertical diameter of the ablation zone on the coronal plane.

^†^Number of HCC nodules. Plus-minus values are means ± standard deviations.

### Complications

No procedure-related death occurred. There were no major complications requiring an increased level of care or more extended hospital stay. One patient from each group had a small amount of pneumothorax, and one patient in the TICW-RFA group showed a small amount of hematoma without evidence of active bleeding. All three patients underwent close observation and were discharged after confirming the decrease of such findings at short-term follow-up CT.

### Technical success, technique efficacy, and clinical outcomes

All patients in both groups showed a technical success. Moreover, at a 1-month follow-up imaging study, the technique efficacy rates were 100% (46/46) in the TICW-RFA group and 95.0% (38/40) in the SC-RFA group (p = 0.213). At the time of the analysis, patients were observed for a mean follow-up of 20.5 months ± 8.3 (median, 23.6 months).

**Fig 3:**
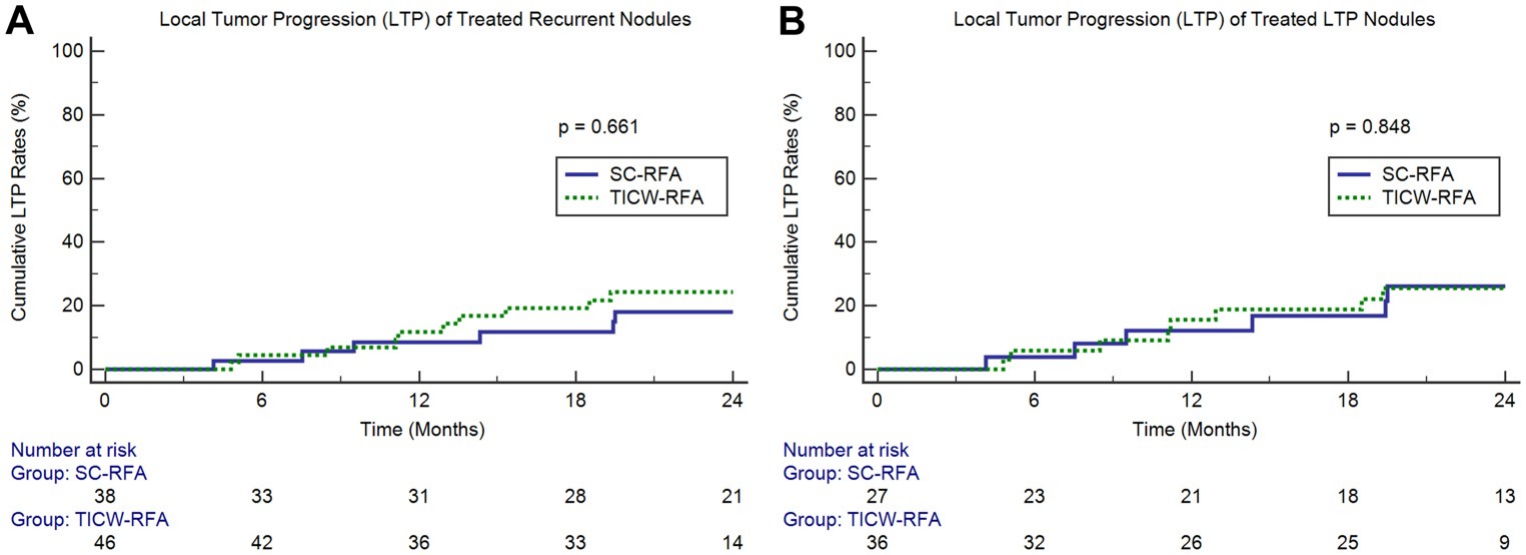
Cumulative LTP rates after RFA of (A) overall recurrent nodules and (B) nodules that presented as LTP.

Out of 46 recurrent HCC nodules treated with TICW-RFA, and 38 with SC-RFA, in which technique efficacy was achieved, cumulative LTP rates at 1 year and 2 years of follow-up were 11.8% and 24.2%, respectively, in the TICW-RFA group, and 8.6% and 18.1%, respectively, in the SC-RFA group (p = 0.661) (Fig 3A). In a subgroup analysis according to the type of recurrence, among 64 nodules that presented as LTP, cumulative LTP rates at 1 year and 2 years after RFA were 15.5% and 25.5%, respectively, in the TICW-RFA group (n = 36) and 12.2% and 26.1%, respectively, in the SC-RFA group (n = 27) (p = 0.848) (Fig 3B).

**Fig 4:**
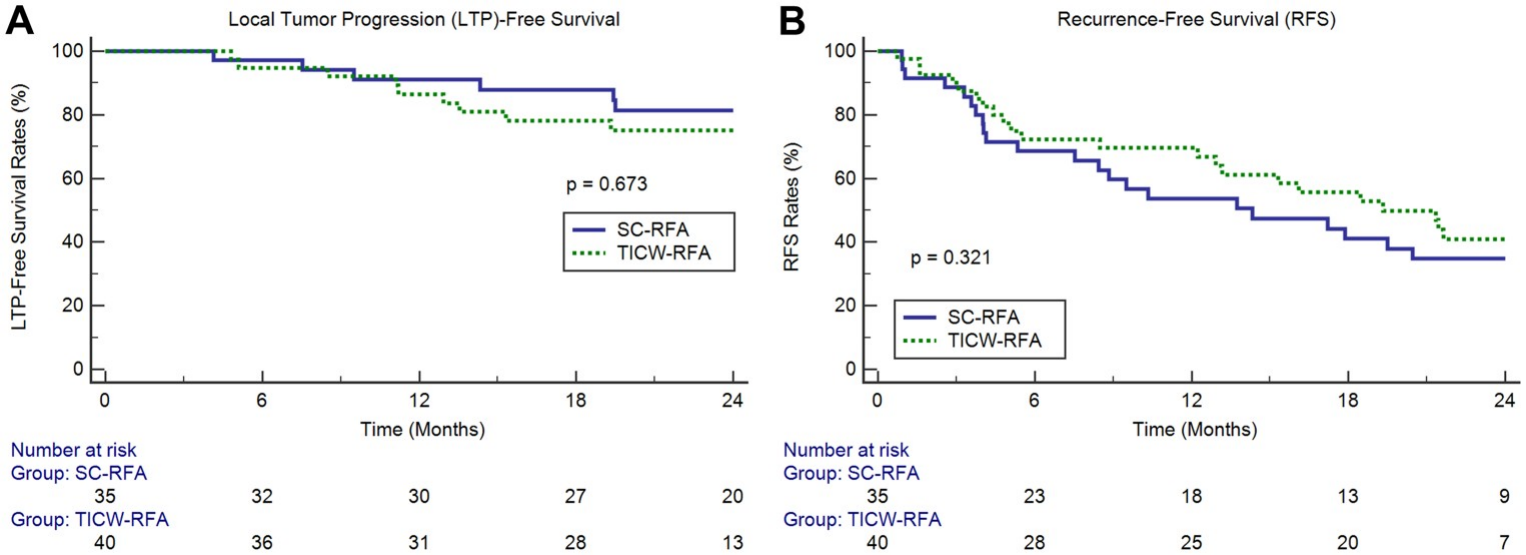
Comparison of clinical outcomes between TICW-RFA and SC-RFA groups. (A) LTP-free survival and (B) RFS in patients treated with TICW-RFA or SC-RFA.

The 1-year and 2-year LTP-free survival rates of 40 patients in the TICW-RFA group were 86.5% and 75.2%, respectively, and those of 35 patients in the SC-RFA group were 91.1% and 81.3%, respectively, (p = 0.673) (Fig 4A). The 1-year and 2-year RFS rates of the patients were 69.6% and 40.8%, respectively, in the TICW-RFA group and 53.7% and 34.7%, respectively, in the SC-RFA group (p = 0.321) (Fig 4B). Moreover, multivariate Cox proportional hazard regression showed that there was no significant prognostic factor for LTP-free survival and only the size of the largest tumor (>1.5 cm; hazard ratio, 2.22; 95% confidence interval, 1.21–4.09; p = 0.010) was a significant prognostic factor for RFS. No AIR occurred in the TICW-RFA group, but one patient in the SC-RFA group developed AIR 19.5 months after the RFA. One patient in the TICW-RFA group died of uncontrolled esophageal variceal bleeding 21.9 months after the RFA procedure.

## Discussion

The present study aimed to compare the two commercially available multiple-electrode RFA systems in Korea: the TICW-RFA and the SC-RFA. From a technical point of view, our study demonstrated that the TICW-RFA group and the SC-RFA group did not show significant differences in the primary endpoint, Dmin per unit time (2.71 ± 0.98 mm/min vs. 2.61 ± 0.96 mm/min, p = 0.577) or ablation time (13.92 ± 4.96 min vs. 15.60 ± 6.80 min, p = 0.441) in patients with recurrent HCCs after locoregional treatments. There were no major complications that involved an elevated level of care or hospital stay in both groups, and they showed high technical success and technique efficacy rates. Therefore, from a clinical point of view, both the TICW-RFA and the SC-RFA were safe and comparably effective treatment choices for the treatment of intrahepatic recurrence of HCC. Our study results regarding Dmin per unit time were discrepant with the results of previous preclinical and clinical studies where RFA, using ICW electrodes, proved more effective in creating large ablation zones [25, 37, 50, 51]. This discrepancy between the previous study and this study, could be mainly attributed to relatively small-sized tumors (average size <2 cm), which may have led to underestimating the theoretical ablative capacities of the two RFA systems and the different number of electrodes between TICW RFA (n = 3) and SC-RFA (n = 2) groups. Nevertheless, the insignificant difference in ablation volumes, despite the geometric disadvantage of using two electrodes, and the lower total RF energy in the TICW-RFA group in our study, supports the better heat-producing efficiency of bipolar RFA with ICW electrodes [25, 37, 50, 51]. In other words, the benefit of bipolar RFA, using TICW in electrical and thermal conductance, compared with switching monopolar RFA, could be canceled out by the negative impact of fewer numbers of active heating sources during the procedure. Furthermore, although there was no difference in major complications between the two groups, there might be a theoretical increased risk of complications, related to electrode insertion, when a higher number of electrodes were used for RFA.

In addition, the 1-year and 2-year cumulative LTP rates in our study were 11.8% and 24.2%, respectively, in the TICW-RFA group and 8.6% and 18.1%, respectively, in the SC-RFA group (p = 0.661); the estimated 2-year LTP-free survival rates for both groups were 75.2% and 81.3% (p = 0.673), respectively. Also, multivariate Cox proportional hazard regression revealed the size of the largest tumor (>1.5 cm; hazard ratio, 2.22; 95% confidence interval, 1.21–4.09; p = 0.010) was the only significant prognostic factor for RFS, which means tumors measuring >1.5 cm have 2.22 times higher risk of overall recurrence than those measuring ≤1.5 cm. The similar LTP rates in the two groups could be attributed to the result that the two groups did not show a significant difference in ablation volumes. Creating a large ablation volume is closely related to a sufficient ablative margin, which is one of the most important factors for local tumor control of HCC [10]. Local tumor control rates in our study were lower than those of RFA for initial HCC (2-year LTP rate, 7%–10%) [38, 39, 52, 53], and somewhat lower than those reported in some previous studies on RFA for recurrent HCC (2-year LTP rate, 10%–25%) [6, 12]. This result was perhaps due to the heterogeneity of our study population, consisting of patients who underwent higher numbers, and various types of previous locoregional treatments. Various off-target effects of locoregional treatments may contribute to the more aggressive potential of recurrent tumors [16–18, 20, 21, 54]. Although RFA has been suggested as a minimally invasive and effective treatment option for recurrent HCC [4–6], the 2-year cumulative LTP rates and RFS rates are reported to be 10%–25% [6, 12] and 20%–43% [6, 13, 55–57], respectively. This high rate of HCC recurrence presents an important clinical challenge, and appropriate treatment is crucial in improving long-term outcomes after treatments [58]. Although many studies have compared different RFA devices and systems in the treatment of initial HCC, similar studies focusing on recurrent tumors are rare; to our knowledge, there is no published randomized controlled study comparing different RFA systems in the treatment of recurrent HCC.

In our study, total RF energy delivery and energy delivery per unit time of the TICW-RFA group were smaller than those of the SC-RFA group, which resulted in a higher minimum diameter per energy in the TICW-RFA group than in the SC-RFA group (p < 0.001). These results of TICW-RFA could be attributed to better concentration of RF energy between the electrodes and also improved electrical conductivity with a saline infusion into the tissue. Electrically, bipolar RFA is able to produce a better concentration of RF energy between the electrodes than monopolar RFA, as it converges energy centripetally from the periphery, while the RF current flows centrifugally in monopolar RFA [33]. Although the disadvantage of conventional bipolar RFA is the possibility of overheating that may lead to charring and insufficient RF energy delivery, cold saline infusion of the ICW electrode used is one way to overcome this problem by preserving thermal and electrical conductivity [59]. Hypothetically, the saline infusion can increase the tissue’s internal pressure that may lead to spreading the cancer cells around the ablation zone, which is one of the proposed mechanisms of AIR [60]; however, no AIR occurred in our TICW-RFA group. In addition to the electrical advantages of bipolar RFA with ICW electrodes, TICW in our study, ICW-RFA using twin electrodes, was able to provide a clinically meaningful advantage of the capability of the “no-touch” technique [61]. Recent studies demonstrated that no-touch RFA in multi-bipolar mode was able to provide better local tumor control for HCC <5 cm than monopolar RFA [52, 62]. Furthermore, Chang et al. [37] recently compared the bipolar RFA and the multi-monopolar RFA, similar to our study, in treatment for small initial HCC, and showed the promising potential of the better tumor control with bipolar RFA. While Chang et al. [37] used switching bipolar mode with three single ICW electrodes, the TICW electrode used in our study, consisted of two active tips and cost the same as one single ICW electrode. Therefore, we believe that bipolar-RFA with TICW electrodes could be a promising method with high-cost effectiveness for obtaining local tumor control for small HCC, compared with monopolar RFA or multi-bipolar RFA with multiple (3–4) electrodes.

In a clinical setting, based on our results, the TICW-RFA and the SC-RFA can be considered comparable treatment options for the treatment of intrahepatic recurrence of HCC, both with similar safety profile and therapeutic efficacy. However, TICW-RFA may help in some clinical scenarios (Table 3). In patients with coagulopathy, a fewer number of electrodes in TICW-RFA may lower the risk of bleeding. TICW-RFA may ensure less thermal damage to adjacent organs for tumors close to the gallbladder or the colon by concentrating the RF energy only between the electrodes. In patients with metallic implants including a pacemaker, electric interference between the device and the RFA system is prevented in TICW-RFA since the RF current flow does not involve a grounding pad but stays only between the electrodes. On the other hand, SC-RFA has geometric advantages over TICW-RFA since it can create an ablation zone according to tumor shape and does not require strict orientation of electrodes.

**Table 3:** Comparison of potential benefits of TICW-RFA and SC-RFA.

	TICW-RFA	SC-RFA
Effect of number of electrodes	Decreases risk of bleeding	Able to create an ablation zone according to shape
Effect of RF system design	No electric interference with metallic implant	Does not require strict orientation of electrodes
	Less adjacent organ injury	
Effect of saline infusion	Improves thermal and electrical conductance in dehydrated tissue	

There are some limitations to our study. First, this study involved a relatively small number of the study population with an intermediate follow-up period. A statistical comparison between the two systems is warranted for a future fully powered randomized controlled trial. Second, as mentioned above, this study included small-sized tumors that may have underestimated the ablative capacities of the two RFA systems. Third, the tumors in our study were most likely heterogeneous in terms of oncological behavior due to relatively broad indications for RFA for the treatment of recurrent HCC in our institution. Although our study, because it was a randomized controlled trial, necessitated reflecting clinical practice as it is, there may be a limitation in generalizing our experience to other institutions or nations.

In conclusion, in this single-center randomized controlled study from a Korean tertiary referral hospital, TICW-RFA demonstrated similar therapeutic efficacy and safety profile for recurrent HCC after locoregional treatment compared with SC-RFA.

## Supporting information

S1 Checklist CONSORT checklist. (DOC)

S1 File Full study protocol in Korean. (DOC)

S2 File Summarized study protocol in English. (DOC)

S3 File Anonymized raw data. (XLSX)
