# Supplementary material for: Radiofrequency ablation using internally cooled wet electrodes in bipolar mode for the treatment of recurrent hepatocellular carcinoma after locoregional treatment: A randomized prospective comparative study
Source: PLoS One. 2020 Sep 28;15(9):e0239733. doi: 10.1371/journal.pone.0239733 (PMC7521687; doi:10.1371/journal.pone.0239733)
Supplement: S2 File — (DOC) [file pone.0239733.s003.doc]

**Radiofrequency Ablation Using Dual Cooled-Wet Electrode for Treatment of Recurred HCCs after Locoregional Treatments: a Preliminary Study**

**Institution: Department of Radiology, Seoul National University Hospital**

**Principal Investigator: Jeong Min Lee**

1. **Study title**Radiofrequency ablation using dual cooled-wet electrodes for treatment of recurred HCCs after locoregional treatments: A preliminary study
2. **Responsible party**
   1. Jeong Min Lee, Department of Radiology, Seoul National University Hospital
   2. Address: Department of Radiology, Seoul National University Hospital, 101 Daehak-ro, Jongno-gu, Seoul, 03080, Korea
3. **Purpose**This study was conducted to provide preliminary data to compare efficacy between switching bipolar radiofrequency ablation (RFA) using dual internally cooled-wet electrodes and switching monopolar RFA using separable clustered electrodes in the treatment of recurrent hepatocellular carcinoma (HCC) after locoregional treatment.
4. **Study design**
   1. Single-center, two-arm, parallel-group, curative-intent, randomized controlled study
   2. Study population
      Patients who satisfy the eligibility criteria and submitted written informed consent were enrolled for a comparative study between bipolar RFA using dual internally cooled-wet electrodes and conventional switching monopolar RFA using separable clustered electrodes in the treatment of recurrent HCC after locoregional treatment. The number of subjects planned to be studied is 80.

      Inclusion criteria
      1. Written informed consent
      2. Aged 20 to 80 years
      3. Patients with underlying liver cirrhosis considered for RFA for treatment of recurrent HCC (1-5 cm) including both local tumor progression and intrahepatic distant recurrence after a locoregional treatment
         ** Diagnostic criteria of recurrent HCC
         1. Pathologic diagnosis of recurrent tumor
         2. Radiologic evidence of recurrent tumor on CT and/or MRI taken within 60 days before the scheduled RFA

Exclusion criteria

1. More than three HCC nodules
2. Tumors with major vascular invasion or abutment to the central portal or hepatic vein with a diameter >5 mm
3. Extrahepatic metastasis
4. Child-Pugh class C
5. Severe coagulopathy (platelet cell count of less than 50,000 cells/mm3 or prothrombin time international normalized ratio (PT-INR) prolongation of more than 50%)
6. A situation where the probability of obtaining appropriate data for the purpose of the study is very low
7. **Expected study duration**
   1. Average number of annual RFA procedures in our institution is about 400. Estimated time for recruitment of 80 patients is one year.
   2. IRB approval ~ 2016.07.31: recruitment and RFA procedure
      IRB approval ~ 2018.01.31: follow-up of subjects
8. **Methodology**
   1. Recruitment
      Patients who meet the eligibility criteria among those who are referred to the radiology department for RFA for recurrent HCC diagnosed by internal medicine physicians and general surgeons
   2. Written informed consent
      Principal investigator provides the purpose of the study, methodology, expected complications, etc. to the patients ahead of the procedure. The subject or representative signs the consent form.
   3. Allocation
      Subjects undergo a 1:1 random assignment to two study arms. A blocked randomization method with mixed block sizes 4 and 6 is performed using a web-based allocation table generated ahead of the study and managed by the institution’s medical research collaboration center. Randomization was stratified by the length of the active tip of the RFA electrode (2 cm or 2.5 cm), as the length of the active tip is determined according to the size of the index tumor.
   4. Treatment planning
      RFA planning is performed as clinical routine protocol. Multiphasic or perfusion CT and Primovist MRI are used to determine the location of the tumor. The US-CT-MR fusion systems of Phillips, GE, or Siemens are used during RFA treatment to confirm the location of the tumor and to monitor the echo bubbles corresponding to the target tumor. Through these fusion systems, the location of the electrodes to be installed on the tumor, the number of electrode insertion, and a safe access route are planned.
   5. RFA ablation
      Two commercially available and currently performed RF systems in our institution were used. In the TICW-RFA arm, bipolar RFA was performed using twin internally cooled-wet electrodes (Jet-Tip Twins, RF Medical, Seoul, Korea) and a single-generator unit (M-3004, RF Medical). In the SC-RFA arm, switching monopolar RFA using separable clustered electrodes with three active tips (Octopus, STARmed, Goyang, Korea) and a dual-generator unit (VIVA Multi, STARmed).
   6. Follow-up
      1. As the clinical routine, CT is performed immediately after RFA to evaluate total necrosis of the target tumor and sufficient ablative margin. If residual masses are identified or safety margins are not secured, additional procedures are performed and evaluated.
      2. One month after RFA, AFP or CEA, CBC, LFT and CT are performed.
      3. Follow-up is performed as clinical routine. Blood test and CT are performed 3 to 4 times for 12 months every 3 months after the procedure. This is due to the fact that most local recurrences occur within 12 months, and the local recurrence rate will be evaluated based on them.
      4. The primary completion date of this study is based on the follow-up findings obtained at 12 months after the procedure (short-term relapse rate evaluation).
      5. However, after 12 months, the patient will continue to perform CT follow-up every 3-6 months until 2 years according to the routine follow-up protocol of RFA.
9. **Outcome evaluation**
   1. Primary endpoint: Dmin per unit time, where Dmin is the shortest diameter of the ablation zone on the axial image with the largest ablation area
   2. Secondary endpoints
      1. Technical parameters including size of the ablation zone, ablation time, and energy delivery
      2. Complication rate
      3. Technical success and technique efficacy
      4. Clinical outcomes including local tumor progression (LTP) rates, LTP-free survival, and recurrence-free-survival
10. **Safety**
    Presence and severity of complications after RFA treatment is evaluated to use the Clavien system
11. **Statistical analysis**
    1. Technical parameters, technical success, technique efficacy, and LTP rates were analyzed with per-nodule data. Complications and other clinical outcomes were analyzed with per-patient data.
    2. Categorical variables were compared using the Chi-squared test or Fisher’s exact test, as appropriate. Continuous variables that did not pass the Shapiro-Wilk normality test were compared using the Mann-Whitney test. Other continuous variables were compared using the independent t-test or Welch test, as appropriate.
    3. Kaplan-Meier method for survival analysis and the log-rank test for assessing differences between the survival curves.
    4. A p-value of less than 0.05 was considered a significant difference.
